# Role of malignant ascites on human mesothelial cells and their gene expression profiles

**DOI:** 10.1186/1471-2407-14-288

**Published:** 2014-04-24

**Authors:** Isabelle Matte, Denis Lane, Dimcho Bachvarov, Claudine Rancourt, Alain Piché

**Affiliations:** 1 Département de Microbiologie et Infectiologie, Faculté de Médecine, Université de Sherbrooke, 3001, 12ième Avenue Nord, Sherbrooke, Québec, J1H 5N4, Canada; 2 Cancer Research Centre, Hôpital L’Hôtel-Dieu de Québec, Centre Hospitalier Universitaire de Québec, 9 rue McMahon, Québec, G1R 2J6, Canada

## Abstract

**Background:**

Malignant ascites is often present at diagnostic in women with advanced ovarian cancer (OC) and its presence is associated with a worse outcome. Human peritoneal mesothelial cells (HPMCs) are key components of malignant ascites. Although the interplay between HPMCs and OC cells is believed to be critical for tumor progression, it has not been well characterized. The purpose of this study was to assess the effect of ascites on HPMCs and clarify the role of HPMCs in OC progression.

**Methods:**

Human OC ascites and benign peritoneal fluids were assessed for their ability to stimulate HPMC proliferation. Conditioned medium from ascites- and benign fluid-stimulated HPMCs were compared for their ability to attenuate apoptosis induced by TNF-related apoptosis-inducing ligand (TRAIL). We conducted a comparative analysis of global expression changes in ascites-stimulated HPMCs using Agilent oligonucleotide microarrays.

**Results:**

As compared to benign peritoneal fluids, malignant ascites stimulated the proliferation of HPMCs. TRAIL-induced apoptosis was attenuated in OC cells exposed to conditioned medium from ascites-stimulated HPMCs as compared to OC cells exposed to conditioned medium from benign fluid-stimulated HPMCs. A total of 649 genes were differentially expressed in ascites-stimulated HPMCs. Based on a ratio of more than 1.5-fold and a P < 0.05, 484 genes were up-regulated and 165 genes were down-regulated in ascites-exposed HPMCs. Stimulation of HPMCs with OC ascites resulted in differential expression of genes mainly associated with the regulation of cell growth and proliferation, cell death, cell cycle and cell assembly and organization, compared to benign peritoneal fluids. Top networks up-regulated by OC ascites included Akt and NF-κB survival pathways whereas vascular endothelial growth factor (VEGF) pathway was down-regulated.

**Conclusions:**

The results of this study not only provide evidence supporting the importance of the interplay between cancer cells and HPMCs but also define the role that the tumor environment plays in these interactions.

## Background

Epithelial ovarian cancer (EOC) is the leading cause of death among gynecological cancers. High-grade serous ovarian carcinomas (HGSOC) are by far the most common (85-90%) subtype and the majority of patients with HGSOC presents with ascites and advanced disease with peritoneal dissemination [1,2]. After initial treatment, the majority of these patients will relapse and eventually die. The mean survival of patients that have advanced disease at presentation is 39 months. This high mortality is mainly attributed to widespread metastasis throughout the peritoneal cavity and the emergence of drug resistance during the course of treatment [3]. OC mortality has not significantly decreased during the last 30 years for reasons including poor understanding of the tumor biology and the interactions with the surrounding environment.

Primary tumor growth induces host responses that are believed to support and promote tumor progression. OC mainly spreads by direct extension, through seeding or exfoliation of tumor cells from ovarian/fallopian tubes into ascites, in which tumor cells survive and proliferate, and later implant in the peritoneal cavity. Indeed, the presence of ascites correlates with intraperitoneal tumor spread and a worse prognosis. In this context, ascites that accumulates during OC progression represent a particular tumor environment and a survival niche for tumor cells [3,4]. Ascites are complex and heterogeneous fluids that contain a variety of cytokines, chemokines and growth factors as well as other soluble factors such as lysophosphatidic acid (LPA) [5,6]. OC tumorigenesis is a complex process and a growing body of evidence suggests that although genetic events in the tumor cells themselves are crucial, host and stromal factors in ascites are also important. For example, OC ascites attenuate drug-induced apoptosis in tumor cells and thus provide a protective environment for tumor cells [4]. Soluble factors in ascites activate survival pathways in tumor cells such as Akt and ERK1\2 signaling, through engagement of cell surface receptors such as αvβ5 integrins which attenuate tumor necrosis factor-related apoptosis-inducing ligand (TRAIL)-induced apoptosis [7-9]. A number of studies have also demonstrated that ascites enhance tumor cell proliferation and migration [10,11]. The presence of LPA in ascites has been shown to promote tumor cell proliferation and migration [12]. These data strongly suggest that malignant ascites plays a significant role in facilitating OC progression and metastasis.

Human peritoneal mesothelial cells (HPMCs) form the peritoneal lining and serve as a protective anatomical barrier. They are among the most abundant cell type in ascites from patients with OC [13]. Although it is becoming evident that paracrine factors secreted in the resulting tumor environment subsequently modify the behaviour of tumor cells, a dynamic interaction between HPMCs found in ascites and the surrounding environment could alter their behaviour, which in turn, further affect malignant evolution and contribute to establish a milieu favouring tumor progression. A number of evidence suggests that morphological and functional changes of HPMCs occur in the presence of cancer cells due to the secretion of paracrine factors. For example, HPMCs increase in size, become more permeable, and undergo an epithelial to mesenchymal transition (EMT) in the presence of TGF-β [14-17]. However, precisely how HPMCs are influenced by ascites is poorly understood.

The aim of this study was to determine the effect of malignant ascites on HPMC behaviour and the paracrine effects of ascites-stimulated HPMCs. We also investigated molecular changes that occur in ascites-stimulated HPMCs. We present evidence that ascites impact on HPMCs by altering their behaviour and gene expression profiles.

## Methods

### Cell culture and clinical samples

The three malignant ascites used in this study (OVC346, OVC508, OVC509) were obtained at the time of initial cytoreductive surgery from three ovarian cancer patients at the Centre hospitalier universitaire de Sherbrooke. Peritoneal fluids were obtained from three patients operated for conditions other than cancer. This study has been performed in accordance with the Declaration of Helsinki and was approved by the «Comité d’éthique de la recherche en santé chez l’humain du centre hospitalier universitaire de Sherbrooke».

Fluids were centrifuged at 1000 rpm for 15 min and the cell-free fractions were stored at -20°C until assayed. All fluids were supplied by the Banque de tissus et de données of the Réseau de Recherche en Cancer of the Fonds de la Recherche du Québec en Santé affiliated to the Canadian Tumor Repository Network (CTRNet). Histopathological diagnosis, grade, and stage of ovarian tumor samples were assigned according to the criteria of the International Federation of Gynecology and Obstetrics. The three malignant ascites were from patients with HGSOC (stage III/IV) and were chosen because they are representative HGSOC ascites with regards to their properties and cytokine profiles [5,7,8]. The ovarian cancer cell lines CaOV3 and SKOV3 were obtained from American Type Culture Collection, (Manassas, VA) and maintained in a humidified 5% CO_2_ incubator at 37°C. Cells were passaged twice weekly. CaOV3 and SKOV3 cells were cultured in DMEM/F12 (Wisent) supplemented with 10% FBS, 2 mM glutamine and antibiotics. HPMCs were isolated from peritoneal lavages of two women operated for conditions other than cancer. After centrifugation, the cell pellet is placed on T25 culture plates. The medium is changed the next day and, in our experience, adhered cells typically represent HPMCs. The nature of HPMCs was confirmed by immunostaining with antibodies against calreticulin (Life Technology) and epithelial marker MOC31 (Oncogen Research Product, San Diego, CA). HPMCs were grown in DMEM/F12 supplemented with 0.4 μg/ml of hydrocortisone and 10 ng/ml EGF (Sigma, Oakville, Canada), 10% FBS and antibiotics. The media was changed every 3 days while the cells were maintained at 37°C in a humidified 5% CO_2_ incubator. HPMCs were used between passage 5-8.

### Immunofluorescence

Cells were grown on glass slides, fixed in cold methanol and blocked in PBS/2% BSA at room temperature for 1 h. Anti-calreticulin and anti-MOC31 primary antibodies were diluted in PBS/BSA and slides were incubated at room temperature for 1 h. Slides were washed twice in cold PBS, incubated 1 h at room temperature either with FITC or Texas-Red conjugated antibodies and visualized with a Olympus IX70 fluorescence microscope (Olympus, Hamburg, Germany).

### In vitro proliferation assay

HPMCs were seeded in medium either with 10% FBS, with 10% benign fluids or with 10% malignant ascites in six-well plates and incubated at 37°C. Cells were monitored for up to 48 h and representative wells were photographed. In some experience, hydroxyurea (30 mM) (Sigma) was added to inhibit cell proliferation. Two independent experiments were performed for each assay and representative photographs were taken. Cell growth was also quantitatively determined using XTT assay as previously described [7].

### RNA preparation and quantitative PCR validation

HPMCs were incubated in medium with either 10% benign fluids or 10% malignant ascites for 4 h. Cells were washed with PBS and total RNA was extracted from HPMCs using TRIzol reagent (Life Technologies) according to the manufacturer’s protocol and subjected to reverse transcription (RT) with oligodT from Promega (Madison, WI) and MMULV reverse transcriptase enzyme. The quality and concentration of RNA was determined by capillary electrophoresis using a Agilent 2100 Bioanalyzer (Agilent Technologies, Mississauga, Ontario, Canada). The integrity of the cDNA was assessed with the Taqman gene expression assays (Life Technologies), done on *18S* housekeeping gene*.* Each sample was normalized to the housekeeping gene levels. For quantitative PCR validation, total RNA was extracted and cDNA was obtained as described above, The FAST Taqman gene expression assay was used with 50 ng of cDNA. Conditions were as follow: initial cycle 50°C, 2 min, 95°C, 10 min. 40 cycles at 95°C, 15 s and 60°C, 1 min on a StepOnePlus^TM^ Real-Time PCR system (Life Technologies). Data were analyzed using the StepOne^TM^ software and comparative ΔΔCt measure was used to express the results as fold changes.

### Gene expression profiling and data analysis

Microarray hybridization was performed using the Whole Human Genome Oligonucleotide Microarray (Agilent), containing ~ 44,000 genes, at the Cancer Research Centre, Hôpital Hôtel-Dieu de Québec. Upon hybridization and washing, the arrays were scanned using a dual-laser DNA microarray scanner (Agilent). The data were extracted from images by the Feature Extraction software 6.1 (Agilent). The GeneSpring software (Agilent) was used to generate lists of selected genes for statistical analysis. An intensity-dependent normalization (Lowess normalization) was applied to correct for artifacts caused by non-linear rates of dye incorporation as well as inconsistencies of the relative fluorescence intensity between dyes. Consecutive lists of differentially expressed genes were generated considering a 1.5-fold expression as the gene selection criteria. The genes in the gene lists were classified according to their function using the Gene Ontology (GO SLIMS) classification system. Network analysis of the microarray data was completed using the Ingenuity Pathway Analysis software (http://www.Ingenuity.com). The microarray data have been deposited to the GEO database (http://www.ncbi.nlm.nih.gov/geo/) with accession number GSE55065.

### Conditioned media and apoptosis assay

To generate HPMC-conditioned media, HPMCs were seeded at 80% density in six-well plates and cultured in media containing either 10% FBS, 10% benign fluids or 10% malignant ascites overnight. Cells were washed twice and fresh medium without FBS or growth factors was added. HPMCs were cultured for 8 to 24 h. Medium conditioned by ascites-stimulated and benign fluids-stimulated HPMCs were applied at a ratio of 50% v/v to CaOV3 cells cultured at 70% density in 12-well plates. CaOV3 cell apoptosis in the presence of TRAIL (25 ng/ml) (PeproTech Inc, Rocky Hill, NJ) was measured using the Cell Death Detection ELISA kit (Roche, Laval, Québec, Canada) according to the manufacturer’s instruction. CaOV3 cells were pre-treated for 1 h with HPMC-conditioned medium before the addition of TRAIL overnight. Three independent sets of experiments were performed for each type of conditioned medium.

### Determination of growth factor levels in ascites

LPA levels in benign peritoneal fluids and malignant ascites were determined by ELISA using the Echelon Biosciences kit (Salt Lake City). TGF-β1 levels were determined using the RayBio® Human Cytokine Antibody Array G series 1000 from RayBiotech Inc. (Norcross, GA). With this method, TGF-β1 levels are expressed as relative fluorescent units (FU) and can be used to compare levels in different ascites. The signal intensities were quantified using the ScanArray Express dual-color confocal laser scanner (Perkin Elmer). Data were collected in Cy3 channel and stored as paired TiFF images. Spots were identified and local background substracted using the TIGR_Spotfinder 3.1.1 software. The internal negative controls were used to determine the cut-off intensity for a positive signal. Intensities up to 750 FU were considered negative.

## Results

### Characterization of mesothelial cultures from the peritoneal lining

**Figure 1:**
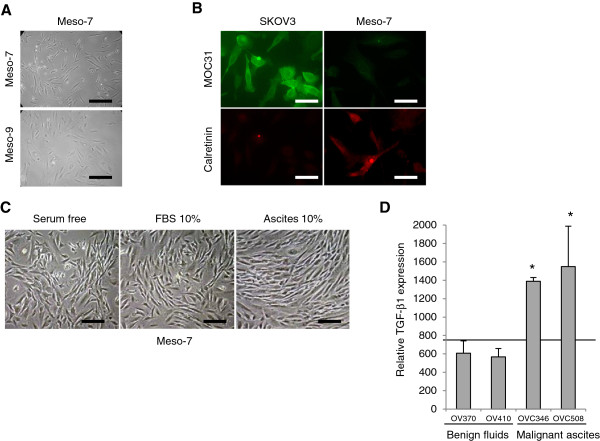
**Characterization of HPMCs. (A)** Phase contrast pictures of HPMCs (Meso-7 and Meso-9) cultured in 10% FBS (x 100 magnification). *Bars* 200 μm. **(B)** Immunofluorescence detection of MOC31 and calretinin in human ovarian cancer cells SKOV3 and HPMCs. The cells were fixed with cold methanol and stained with FITC-conjugated anti-MOC31 and Texas Red-conjugated anti-calretinin (x 1000 magnification). HPMCs stained positive for calretinin and negative for MOC31 confirming that they were mesothelial cells. *Bars* 30 μm. **(C)** HPMCs were cultured either with absence of FBS, 10% FBS or 10% malignant ascites (OVC508) and representation phase contrast images were taken (x 200 magnification). *Bars* 100 μm. **(D)** The relative expression of TGF-β1 was determined as described in Material & Methods for peritoneal benign fluids (OV370 and OV401) and malignant ascites (OVC346 and OVC508). The solid line indicates the cut-off intensity (750 FU) for a positive signal. *indicate *P* < 0.001, T-student test.

We established HPMC cultures of peritoneal fluids from two women with benign conditions. The morphology of two primary HPMC samples (Meso-7 and Meso-9) cultured in presence of 10% FBS is shown in Figure 1A. These cells show spindle fibroblastic-like pattern consistent with a mesenchymal phenotype. The primary HPMC cultures of Meso-7 were further characterized using MOC31 epithelial marker and calretinin mesothelial marker [18]. As shown in Figure 1B, mesothelial cultures stained positive for calretinin and negative for MOC31 as expected, confirming the absence of epithelial cells in HPMCs. In contrast, the SKOV3 OC cell line stained positive for MOC31 and negative for calretinin. Furthermore, as previously reported [14], HPMCs cultured in serum-free medium exhibited a polygonal, even cobblestone-like morphology (Figure 1C). In contrast, HPMCs cultured in 10% malignant ascites exhibited a more fibroblastic-like pattern.

Because TGF-β1 has been previously associated with morphologic changes in HMPCs [14], we examined the levels of TGF-β1 from benign fluids and malignant ascites. Interestingly, the levels of TGF-β1 were significantly higher (*P* < 0.001) in malignant ascites compared to benign fluids (Figure 1D). TGF-β1 levels were below the threshold for positivity (750 FU) in the two benign peritoneal fluids tested.

### Malignant ascites stimulate the growth of HPMCs

**Figure 2:**
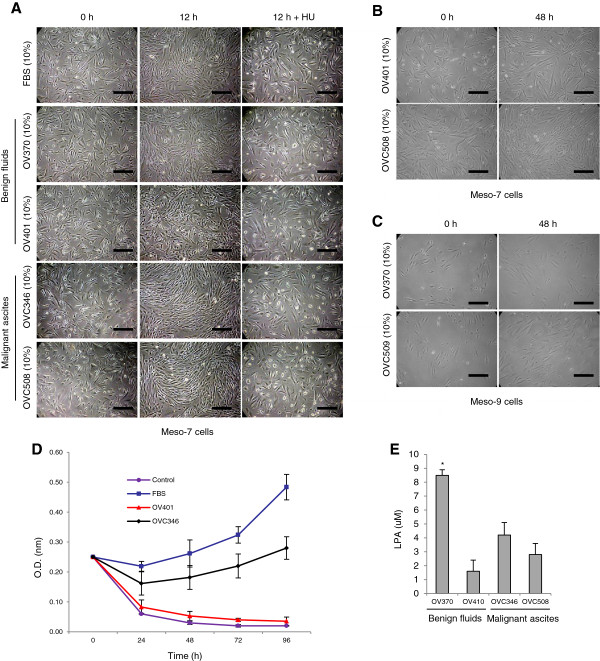
**Effect of ascites on HPMC proliferation. (A)** HPMCs were cultured either with 10% FBS, 10% benign peritoneal fluids or 10% malignant ascites in the presence or absence of hydroxyurea (HU) for 12 h and phase contrast images were taken (x 200 magnification). *Bars* 100 μm. **(B-C)** Phase contrast pictures of Meso-7 and Meso-9 cells cultured either with 10% benign fluid (OV370 or OV401) or 10% malignant ascites (OVC508 or OVC509). *Bars* 100 μM. **(D)** HPMC (meso-7 cells) were seeded and cell growth for up to 96 h was determined by XTT assay. **(E)** Determination of LPA levels in benign fluids or malignant ascites. There was no significant difference between levels of LPA in OV401, OVC508 and OVC509 (P > 0.05). Levels of LPA in OV370 were however significantly higher. *indicate *P* < 0.01, T-student test.

Malignant ascites constitute a dynamic reservoir of soluble factors, which individually and in a combined fashion may affect cell behavior. To assess the putative effect of malignant ascites on the growth of HPMC cultures, we selected two representative ascites (OVC346 and OVC508) obtained from women with newly diagnosed HGSOC. These malignant ascites have been previously described [5,7,8]. This study included only HGSOC ascites because they are the most clinically relevant as the majority of patients presenting with ovarian cancer (80-90%) have HGSOC. HPMCs were incubated with OVC346 and OVC508 cell-free ascites fractions and two peritoneal fluids from women with benign gynecological conditions. Compared to the peritoneal benign fluids, a growth-enhancing effect was observed with the two malignant ascites as shown by an increased in overall cell number after 12 h (Figure 2A). Both OVC346 and OVC508 malignant ascites had growth-enhancing activity compared to benign fluids. The growth-enhancing effect of malignant ascites was completely inhibited by the addition hydroxyurea, a cell cycle inhibitor. When compared to benign fluid OV401, a growth-enhancing activity on HPMCs was observed for up to 48 h with malignant ascites (Figure 2B). To ensure that the effect of ascites was not limited to a single HPMC culture, we also tested the effect of ascites on Meso-9 mesothelial culture. Malignant ascites (OVC509) also enhanced the growth of Meso-9, although these cells grew at a much slower rate than the Meso-7 cells suggesting that the effect of malignant ascites on growth is reproducible in different HPMC culture (Figure 2C). The cell growth of HPMCs in the presence of benign fluid (OV401) and malignant ascites OVC346 was also monitored by XTT assay and demonstrated that OVC346 stimulated cell growth whereas OV401 did not (Figure 2D). These data suggest that ascites contain soluble factors that stimulate the proliferation of the two patient-derived HPMC cultures.

LPA is a growth factor-like phospholipid present in the serum and ascites of patients with OC and promotes tumor cell proliferation [6]. LPA has been reported to be present at higher concentration in malignant ascites when compared to benign fluids [6]. However, we found that LPA levels were not consistently higher in malignant ascites OVC346 and OVC508 when compared to benign fluids (Figure 2E). A more extensive analysis of LPA levels in benign fluids (n = 17) versus serous OC (n = 20) also failed to show higher levels of LPA in serous OC (median 1.9 μM ± 1.1 for benign fluids versus 3.0 μM ± 1.9 for serous OC; *P* > 0.05).

### Malignant ascites-stimulated HPMCs secrete soluble factors that attenuate TRAIL-induced apoptosis

**Figure 3:**
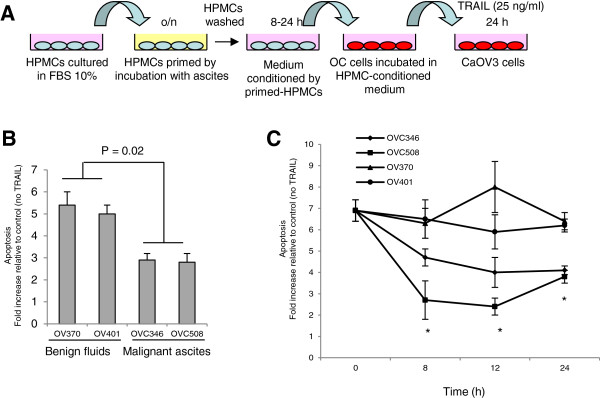
**TRAIL-induced apoptosis in ascites-stimulated HPMCs. (A)** Diagram of HPMC-priming assays. Ascites-stimulated or benign fluid-stimulated HPMCs were culture overnight (shown in yellow), washed TWICE and cultured in serum/hormone-free medium for 8 to 24 h to generate HPMC-conditioned medium (shown in pink) that were collected at either 12 h **(B)** or different time points **(C)**. HPMC-conditioned medium was then added to CaOV3 tumor cells in the presence of TRAIL (25 ng/ml). TRAIL-induced apoptosis was measured in CaOV3 cells incubated with the indicated HPMC-conditioned medium overnight and expressed as fold increased relative to cells that were exposed to HPMC-conditioned medium but not to TRAIL. Data are expressed as means of triplicates from three independent experiments ± SD. * indicate *P* < 0.01.

Soluble factors produced by cancer-associated fibroblasts and bone marrow stromal cells have been shown to confer resistance to TRAIL-induced apoptosis in tumor cells [19-21]. We reasoned that malignant ascites-stimulated HPMCs might also secrete soluble factors that could attenuate TRAIL-induced apoptosis. HPMCs were incubated with benign fluids or malignant ascites overnight. The cells were then washed twice and conditioned media (CM) were collected 12 h later. Ovarian cancer CaOV3 cells were treated with TRAIL in presence of CM from HPMCs exposed to either benign fluids or malignant ascites and apoptosis was measured (Figure 3A). As shown in Figure 3B, TRAIL-induced apoptosis was decreased in CaOV3 cells exposed to CM from malignant ascites-exposed HPMCs as compared to CM from benign fluid-exposed HPMCs. These results suggest that ascites-stimulated HPMCs secrete soluble factors that attenuate TRAIL-induced apoptosis. To examine the effect of ascites exposure on the secretion of soluble factors overtime, HPMCs were stimulated with malignant ascites or benign fluids overnight. Cells were then washed twice and CM were collected after 8, 12 and 24 h. Whereas CM from benign fluid-stimulated HPMCs collected at different time did not affect TRAIL-induced apoptosis (OV370, OV401), CM from ascites-stimulated HPMCs significantly reduced apoptosis in CaOV3 cells (Figure 3C). The maximum protection was observed at 12 h.

### Gene expression changes induced by malignant ascites

**Figure 4:**
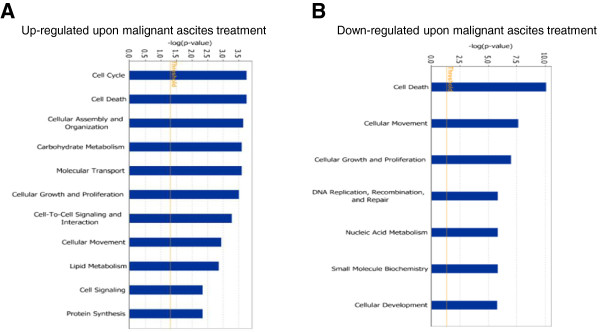
**Functional analysis for the dataset of differentially expressed genes (≥ 1.5-fold) in ascites-stimulated HPMC cells.** Top functions that meet a *P* value cutoff of 0.05 are displayed. The orange line represents the cutoff value for significance. **(A)** Genes that were up-regulated and **(B)** genes down-regulated.

**Figure 5:**
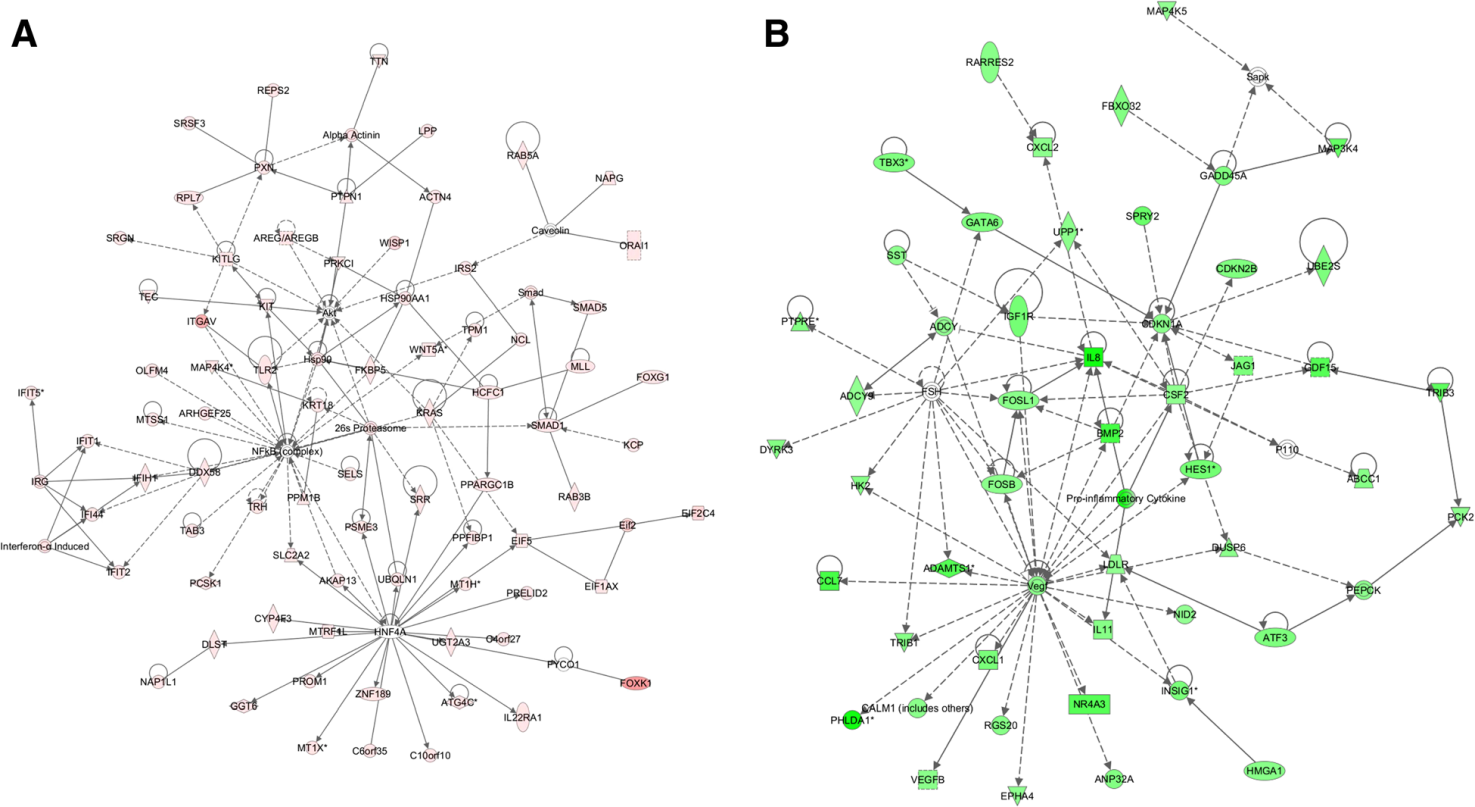
**Network analysis of dynamic gene expression in ascites-stimulated HPMCs based on the common up-regulated (A) or down-regulated (B) (≥ 1.5-fold) gene expression list obtained following stimulation with all three malignant ascites.** The top-scoring networks were merged and displayed graphically as node (gene/gene product) and edges (the biological relationships between the nodes). Nodes are displayed using various shapes that represent the functional class of the gene product (square, cytokine; vertical oval, transmembrane receptor; rectangle, nuclear receptor; diamond, enzyme; rhomboid, transporter; hexagon, translation factor; horizontal oval, transcription factor; circle, other). Edges are displayed with various labels that described the nature of relationship between the nodes: — binding only; → acts on. The length of an edge reflects the evidence supporting that node-to-node relationship, in that edges supported by article from literature are shorter. Dotted edges represent indirect interaction.

The expression profiles from HPMC cultures exposed to peritoneal fluids and OC ascites were compared using the Whole Human Genome Oligonucleotide Microarray (Agilent), containing ~ 44,000 genes. Microarrays were performed on HPMCs exposed to 3 malignant ascites from women with advanced (stage III/IV) serous OC and two benign peritoneal fluids. First, we generated lists of significantly up-regulated and down-regulated genes that were differentially expressed between OC ascites (OVC346, OVC508 and OVC509) and control OV370 peritoneal fluid. Then, the set of genes that were commonly expressed between control peritoneal fluids (OV370 and OV401) were subtracted from the first list of genes to generate a dataset of differentially expressed genes between malignant ascites and benign peritoneal fluids. A subset of 649 genes was thus selected by filtering on confidence at *P* value = 0.05, followed by filtering on expression levels (≥ 1.5 fold). We found 484 genes to be commonly up-regulated and 185 genes to be down-regulated in HPMCs exposed to malignant ascites. Top molecules that were up-regulated are shown in Table 1 and those down-regulated in Table 2. Pathway and network analysis based on the 649 genes list were generated through the use of Ingenuity Pathways Analysis (IPA). IPA showed that the top two pathways up-regulated in this gene list were functionally associated with the regulation of cell cycle and apoptosis (Figure 4A) which is consistent with data from Figures 2 and 3. Genes implicated in cell death and cell growth and proliferation (mostly negative regulators) were among the top pathways down-regulated (Figure 4B) (Table 3). Networks linked to cancer, inflammatory response, cell movement, cell assembly and organization, cell-to-cell signaling, DNA replication, and repair and recombination were both induced or suppressed. The analysis recognized several important nodes linked with numerous partners, including nuclear factor-κB (NF-κB), Akt, heat shock protein 90 (Hsp90), hepatocyte nuclear factor 4α (HNF-4α), KRAS, SMAD1, RNA helicase p68 (DDX5B, p68), c-KIT ligand (KITLG), vascular endothelial growth factor (VEGF), interleukin-8 (IL-8), follicle stimulating hormone (FSH), colony stimulating factor 2 (CSF2), cyclin-dependent kinase inhibitor 1A (CDKN1A, p21, Clip1), bone morphogenetic protein 2 (BMP2) (Figure 5A and B). While some of the up-regulated gene nodes and related pathways were associated with positive feedbacks on the cell cycle (NF-κB, KRAS, KITLG, MAP4K4, HCFC1), some down-regulated genes were negative regulators of the cell cycle (CDKN1A/2B, DUSP6/10).

**Table 1:** **Up-regulated genes in HPMCs exposed to malignant ascites versus to benign fluids (Fold change > 2, *P*** **< 0.05)**

***Unigene***	***Symbol***	***Description***	***Functions***	***Fold change***
Hs.425633	*CKMT1B*	Creatine kinase mitochondrial 1B	Mitochondria	31.80
Hs.282409	*CYP2C19*	Cytochrome P450, family 2, subfamily C	Electron transport	29.10
Hs.560	*APOBEC1*	Apolipoprotein B mRNA editing enzyme, catalytic polypeptide 1	mRNA processing	17.19
Hs.516922	*NKX2-2*	NK2 transcription factor related locus 2	Transcription	16.20
Hs.553484	*ANGPT2*	Angiopoietin-2A	Angiogenesis	16.16
Hs.145932	*MTL5*	Metallothionein-like 5	Anti-apoptotic	11.40
Hs.445098	*DEPDC1*	DEP domain containing 1	Intracellular signaling	7.06
Hs.149924	*LILRB1*	Leucocyte immunoglobulin-like receptor, subfamily B	Immune response	6.31
Hs.471494	*EIF2C4*	mRNA for KIAA1567	Protein synthesis	6.06
Hs.272215	*KLF15*	Kruppel-like factor 15	Transcription	5.74
Hs.285829	*DLEU7*	Deleted in lymphocytic leukemia 7	Intracellular signaling	5.23
Hs.6702	*KCNB2*	Potassium voltage-gated chanel	Cation transport	5.08
Hs.351403	*DZIP1L*	DAZ interacting protein 1-like	Ion binding	4.60
Hs.479853	*EPHA5*	EPH receptor A5	Intracellular signaling	4.54
Hs.122583	*UTG2A3*	UDP glucuronosyltransferase 2 family	Metabolism	4.23
Hs.446021	*SLC37A3*	Solute carrier family 37	Transport	4.02
Hs.270833	*AREG*	Amphiregulin	Cell proliferation	3.52
Hs.467793	*FLJ40869*	Hypothetical protein FLJ40869	DNA repair	3.31
Hs.441975	*BIRC4BP*	XIAP-associated factor 1	Ion binding	3.26
Hs.531941	*MYB*	myeloblastosis viral oncogene homolog	Transcription	2.83
Hs.585869	*ZNF224*	Zinc finger protein 224	Transcription	2.57
Hs.188518	*MT1K*	Metallothionein 1 K	Ion binding	2.54
Hs.190043	*MOSPD2*	Motile sperm domain containing 2	Membrane protein	2.52
Hs.270543	*GNB4*	Guanine nucleotide binding protein, beta polypeptide 4	Intracellular signaling	2.44
Hs.190622	*DDX58*	DEAD box polypeptide 58	DNA binding	2.37
Hs.658169	*SFRP4*	Secreted frizzled-related protein 4	Intracellular signaling	2.37
Hs.13852	*DNAJB4*	Hsp40 homolog, subfamily B, member 4	Protein folding	2.36
Hs.189920	*PB1*	Polybromo 1	Transcription	2.35
Hs.513044	*CSPG4*	Chondroitin sulfate proteoglycan 4	Cell motility	2.32
Hs.520506	*FBXO5*	F-box protein 5	Protein degradation	2.28
Hs.83634	*HCFC1*	Host cell factor C1	Cell proliferation	2.25
Hs.14794	*ZFP28*	Zinc finger protein 28 homolog	Transcription	2.19
Hs.10319	*UGT2B7*	UDP glucuronosyltransferase 2 family	Metabolism	2.17
Hs.503093	*ZFP36L2*	Zinc finger protein 36, C3H type-like 2	Cell proliferation	2.14
Hs.110915	*IL22RA1*	Interleukin 22 receptor, alpha 1	Immune response	2.13
Hs.442344	*IRS2*	Insulin receptor substrate 2	Cell proliferation	2.11
Hs.479670	*TEC*	Tec protein tyrosine kinase	Intracellular signaling	2.10
Hs.167700	*SMAD5*	SMAD, family member 5	Transcription	2.10
Hs.541894	*ANKRD36*	Ankirin repeat domain 36	Catalytic activity	2.09
Hs.655199	*MT1A*	Metallothionein 1A	Ion binding	2.09
Hs.407190	*FKBP5*	FK506 binding protein 5	Protein folding	2.06
Hs.744289	*ZNF549*	Zinc finger protein 549	Ion binding	2.06
Hs.431550	*MAP4K4*	Mitogen-activated protein kinase kinase kinase kinase 4	Intracellular signaling	2.04
Hs.433702	*EIF5*	Eukaryotic translation initiation factor 5	Translation	2.04
Hs.167584	*SLC2A2*	Solute carrier family 2	Transport	2.03
Hs.405144	*SFRS3*	Splicing factor, arginine/serine-rich 3	RNA binding	2.02

**Table 2:** **Down-regulated genes in HPMCs exposed to malignant ascites versus to benign fluids (Fold change > 2, *P*** **< 0.05)**

***Unigene***	***Symbol***	***Description***	***Functions***	***Fold change***
Hs.602085	*PHLDA1*	Pleckstrin homology-like domain, member 1	Nuclear protein	-3.40
Hs.624	*IL-8*	Interleukin 8	Angiogenesis	-3.38
Hs.98367	*SOX17*	SRY-box 17	Transcription	-3.33
Hs.345139	*GEM*	GTP binding protein overexpressed in skeletal muscle	Intracellular signaling	-2.58
Hs.73853	*BMP2*	Bone morphogenetic protein 2	Cell proliferation	-2.58
Hs.251526	*CCL7*	Chemokine ligand 7	Inflammatory response	-2.54
Hs.591159	*PTHLH*	Parathyroide hormone-like hormone	Ion binding	-2.44
Hs.643357	*ADAMTS1*	A disintegrin and metallopeptidase with thrombospondin type 1	Cell proliferation	-2.34
Hs.517310	*RIPK4*	Receptor-interacting serine-threonine kinase 4	Intracellular signaling	-2.32
Hs.616962	*GDF15*	Growth differentiation factor 15	Intracellular signaling	-2.31
Hs.279522	*NR4A3*	Nuclear receptor subfamily 4, group A, member 3	Transcription	-2.30
Hs.25829	*RASD1*	RAS, dexamethasone-induced 1	Intracellular signaling	-2.27
Hs.516826	*TRIB3*	Tribbles homolog 3	Transcription	-2.22
Hs.406714	*KRTAP2-4*	Keratin associated protein 2-4	Cell structure	-2.20
Hs.505146	*CLDN14*	Claudin 14	Cell-cell adhesion	-2.20
Hs.799	*HBEGF*	Heparin-binding EFG-like growth factor	Growth factor	-2.18
Hs.525572	*BDKRB1*	Bradykinin receptor B1	Inflammatory response	-2.18
Hs.191215	*PSCD1*	Pleckstrin homology, Sec7 and coiled-coil domains	Transport	-2.07
Hs.76095	*IER3*	Immediate early response 3	Apoptosis	-2.04
Hs.18676	*SPRY2*	Sprouty homolog 2	Cell-cell signaling	-2.03

**Table 3:** **Cell death biological function category showing a significant fold change in genes belonging to HPMCs exposed to malignant ascites compared to benign fluids (Fold change > 2, *P*** **< 0.05)**

***Symbol***	***Description***	***Fold change***
*BDKRB1*	Bradykinin receptor B1	-2.18
*IL-8*	Interleukin 8	-3.38
*SOX17*	SRY-box 17	-3.33
*BMP2*	Bone morphogenetic protein 2	-2.58
*NR4A3*	Nuclear receptor subfamily 4, group A, member 3	-2.30
*RASD1*	RAS, dexamethasone-induced 1	-2.27
*PHLDA1*	Pleckstrin homology-like domain, member 1	-3.40
*TRIB3*	Tribbles homolog 3	-2.22
*HBEGF*	Heparin-binding EFG-like growth factor	-2.20
*SPRY2*	Sprouty homolog 2	-2.03
*AREG*	Amphiregulin	3.52
*DDX58*	DEAD box polypeptide 58	2.37
*DEPDC1*	DEP domain containing 1	7.06
*IRS2*	Insulin receptor substrate 2	2.11
*SMAD5*	SMAD, family member 5	2.10
*MT1A*	Metallothionein 1A	2.10
*MAP4K4*	Mitogen-activated protein kinase kinase kinase kinase 4	2.04

### Validation of microarray findings with quantitative RT-PCR

**Figure 6:**
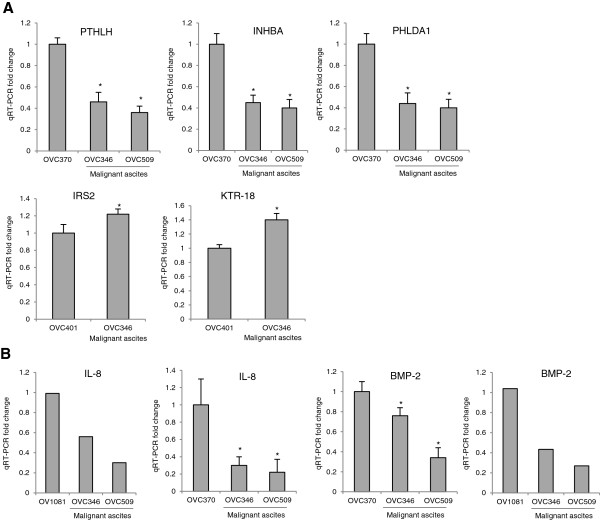
**Correlation between gene expression data and quantitative PCR.(A)** Levels of mRNA expression in ascites-stimlated HPMCs expressed as fold changes relative to benign fluiids OVC370- or OVC401-stimulated HPMCs. **(B)** Relative expression of IL-8 and BMP-2 mRNA in ascites-stimulated HPMCs compared to either benign fluid OV1081- or OVC370-stimulated HPMCs. * indicate *P* < 0.03.

To validate the results of the microarray analysis, we used quantitative real-time PCR to quantify the expression of selected genes including PTHLH, INHBA, PHLDA1, IRS2 and KTR-18 in ascites-stimulated HPMCs compared to benign fluid (OVC370, OVC401)-stimulated HPMCs. qRT-PCR analysis confirmed our microarray findings for PTHLH, INHBA and PHLDA1 genes which were down-regulated, and for IRS2 and KTR-18 which were up-regulated (Figure 6A). qRT-PCR analysis was also performed with a third peritoneal fluid OV1081 along with OV370 to validate the differential expression of IL-8 and BMP2 in malignant ascites (Figure 6B). The expression of IL-8 and BMP2 were down-regulated in HPMCs stimulated with malignant ascites as compared to both OV1081 and OV370 benign fluids.

## Discussion

A crucial step in OC progression is the ability of tumor cells to shed from the primary tumor site and proliferate and survive in ascites. In this context, ascites must provide a milieu that support tumor cell growth. OC ascites are rich, heterogeneous and complex fluids that harbor a wide variety of soluble factors that are part of an autocrine and paracrine network in tumor cells. In line with these observations, the presence of ascites correlates with peritoneal spread of OC tumors [22] and significantly decreases the 5-year survival rate for women with advanced OC [23]. Malignant ascites offer OC cells a network of proliferative and survival factors; thus OC cells floating in ascites receive signals that alter gene expression which confer a survival advantage. Indeed, it was recently demonstrated that ascites promote the activation of survival pathways in tumor cells, which contribute to attenuate drug-induced apoptosis [7-9]. Changes in tumor cell behavior are mediated by the activation of various signaling pathways such as PI3K/Akt and MAPK/ERK pathways in these cells [8,9]. HPMCs present in ascites are theoretically exposed to those same factors and consequently receive similar signals. To better understand the role of HPMCs in OC progression and how ascites signals may alter their behavior, we characterized the effects of malignant ascites on HPMC morphology and proliferation, and correlated these effects with molecular alterations in gene expression occurring in HPMCs after exposure to malignant OC ascites. We used low passage two patient-derived HPMC cultures that were derived from peritoneal fluids and exposed these cells to either malignant ascites or benign peritoneal fluids. We analyzed functionally related genes that were commonly differentially expressed following exposure of HPMCs to all malignant ascites compared to benign peritoneal fluids.

The current study demonstrates that OC ascites consistently induce a switch of morphology in HPMCs from an epithelial to a fibroblastic pattern, a finding that has been reported by other groups when HPMCs were incubated with TGF-β1 [14,15]. In contrast, benign fluids failed to induce such a switch. Interestingly, levels of TGF-β1 were below the threshold of positivity in benign fluids whereas TGF-β1 was detectable in malignant ascites, although levels were low (~ 1400 FU). TGF-β1 is considered a critical regulator of epithelial-to-mesenchymal transition (EMT). The essential features of EMT include the downregulation of epithelial cell markers (*e.g.* E-cadherin, claudins) and the upregulated expression of fibroblastic markers (*e.g.* vimentin, N-cadherin). TGF-β1-induced EMT is mediated by Smad-dependent and –independent signaling [24]. Whether the low level of TGF-β1 found in malignant ascites is responsible for the morphologic changes that were observed in HPMCs is unclear. Smad1 and Smad5 genes were up-regulated by malignant ascites which is consistent with the involvement of TGF-β1. Signaling pathways involved in EMT such as PI3K/Akt and Ras/MAPK were also up-regulated by malignant ascites (Figure 5A). All these findings are consistent with an important role for TGF-β1. However, growth factors other than TGF-β1, such as hepatocyte growth factor (HGF), fibroblast growth factor (FGF) or epidermal growth factor (EGF), which are found in malignant ascites [5], may also activate these signaling pathways and induce EMT [24]. In the current study, we observed that the three OC ascites tested stimulated the proliferation of HPMCs. In contrast, the two peritoneal fluids did not stimulate proliferation. This suggests that the malignant ascites tested contain growth-promoting activity. In line with this observation, malignant ascites were also found to stimulate the proliferation of OC cells *in vitro*[10]. Malignant ascites contain several growth factors that could potentially stimulate the proliferation of mesothelial cells [5]. Among these factors, LPA is of particular interest. In the present study, we showed that LPA is detectable in both malignant ascites and in benign fluids (Figure 2E). It has been previously reported that LPA is present at 20-80 μM concentrations in the ascites of OC patients [25-27]. LPA is a factor in ascites from OC patients that promote the proliferation as well as the migration of OC cells [28-30]. Serous OC LPA levels were lower in this study (median 3.0 μM) compared to previous studies. Most importantly however, the levels of LPA were not significantly different in serous OC compared to benign fluids. These observations suggest that, in the two malignant ascites tested, LPA may not be a critical factor for ascites-mediated proliferation of the two samples of HPMCs.

Consistent with the findings that malignant ascites stimulate HPMC proliferation *in vitro*, we found that cell cycle- and cell growth-related genes were up- and down-regulated by malignant ascites. In total, the expression of 85 genes involved in cell proliferation was altered by malignant ascites. In particular, several cyclin-dependent kinase (Cdk) inhibitors (p21, p15) and dual specificity phosphatases (DUSP6, DUSP10) were down-regulated. Upon stimulation by growth factors, downstream targets such as cyclin D1 are activated by the ERK pathway, which is activated by LPA [30], resulting in progression from G1 to S. Cdks inhibitors such as p21 and p15 can block G1 progression. Dusp6 and Dusp10 acts as negative feedback regulators of ERK signalling [24,31]. Conversely, genes such as receptor tyrosine kinase KIT, its ligand stem cell factor (SCF) and KRAS, which induce ERK phosphorylation and promote cell proliferation [32], were upregulated by ascites.

Our data indicate that the two OC ascites tested induce the secretion of factors by HPMCs (at least in the meso-7 samples) that attenuate TRAIL-induced apoptosis in tumor cells. This observation implies that ascites activate HPMCs through paracrine interactions and activated HPMCs secrete factors that promote the survival of tumor cells. Indeed, many genes differentially expressed in HPMCs stimulated by malignant ascites are closely related to the regulation of apoptosis. The apoptosis-related genes include a total of 47 genes that were down-regulated and 58 that were up-regulated (Figure 4). Interestingly, stem cell factor (SCF) and its receptor (c-kit) were among the genes that were up-regulated. Myb transcription factor, which serves as a regulator of c-kit expression, was up-regulated by ascites in HPMCs. SCF/c-kit pathway has been implicated in a variety of processes including cell survival [33]. SCF signals via c-kit through PI3K/Akt and Ras/MAPK pathways, two well-establish survival pathways [33]. Ahmed et al. showed that ascites activate Ras/MAPK signaling in OC cells [34]. Our group also demonstrated that OC ascites stimulate MAPK/ERK1/2 pathway leading to the regulation of Mcl-1 antiapoptotic protein in OC cells [9].

## Conclusions

In summary, this study provides evidence that activation of HPMCs is mediated by paracrine interactions with soluble factors in malignant ascites. These factors stimulate a phenotypic shift from an epithelial to a fibroblastic morphology in HPMCs. Ascites-stimulated HPMCs are proliferative and secrete soluble factors that promote tumor cell survival. Although the nature of these factors remains to be determined, they likely promote a survival advantage for tumor cells. Paracrine factors in ascites activate intracellular signaling network such as Akt and NF-κB in HPMCs which mediate, in turn, the up-regulation of HPMC-secreted factors that impact OC progression. One limitation of this study is that data were derived from a small number of samples, thus conclusions should be viewed appropriately. Validation in a larger set of patients will be beneficial. Future studies assessing the nature of paracrine and autocrine stimulating signals will help to better define the interplay between HPMCs and tumor cells that is important for OC progression.

## Competing interests

The authors declare that they have no competing interest.

## Authors’ contributions

IM participated in the design of the study and performed all functional assays. IM was also responsible for obtaining the clinical samples. DL performed the LPA measurements in the clinical samples. DB performed the microarray data analysis. CB performed the characterization of HPMCs by immunofluorescence. CR participated in the design of the study and helped to draft the manuscript. AP conceived the study, participated in its design and drafted the manuscript. All authors read and approved the final manuscript.

## Pre-publication history

The pre-publication history for this paper can be accessed here:

http://www.biomedcentral.com/1471-2407/14/288/prepub
